# Molecular and functional analysis of monoclonal antibodies in support of biologics development

**DOI:** 10.1007/s13238-017-0447-x

**Published:** 2017-07-21

**Authors:** Xin Wang, Zhiqiang An, Wenxin Luo, Ningshao Xia, Qinjian Zhao

**Affiliations:** 1 State Key Laboratory of Molecular Vaccinology and Molecular Diagnostics, National Institute of Diagnostics and Vaccine Development in Infectious Diseases, School of Public Health, Xiamen University, Xiamen, 361105 China; 2 School of Life Sciences, Xiamen University, Xiamen, 361105 China; 3 Texas Therapeutics Institute, The Brown Foundation Institute of Molecular Medicine, The University of Texas Health Science Center at Houston, Houston, TX 77054 USA

**Keywords:** monoclonal antibody, molecular characterization, ligand binding assay, cell based assay, heterogeneity, functional assessment

## Abstract

Monoclonal antibody (mAb)-based therapeutics are playing an increasingly important role in the treatment or prevention of many important diseases such as cancers, autoimmune disorders, and infectious diseases. Multi-domain mAbs are far more complex than small molecule drugs with intrinsic heterogeneities. The critical quality attributes of a given mAb, including structure, post-translational modifications, and functions at biomolecular and cellular levels, need to be defined and profiled in details during the developmental phases of a biologics. These critical quality attributes, outlined in this review, serve an important database for defining the drug properties during commercial production phase as well as post licensure life cycle management. Specially, the molecular characterization, functional assessment, and effector function analysis of mAbs, are reviewed with respect to the critical parameters and the methods used for obtaining them. The three groups of analytical methods are three essential and integral facets making up the whole analytical package for a mAb-based drug. Such a package is critically important for the licensure and the post-licensure life cycle management of a therapeutic or prophylactic biologics. In addition, the basic principles on the evaluation of biosimilar mAbs were discussed briefly based on the recommendations by the World Health Organization.

## Introduction

Orthoclone OKT3^®^ (muromonab-CD3), the first therapeutic monoclonal antibody, was approved in 1985 by the U.S. Food and Drug Administration as an antirejection agent for renal transplantation (Goldstein, 1987; Smith, 1996). Since then, the development of monoclonal antibodies (mAbs) as therapeutic drugs has become a hot area in biopharmaceutical industries (Pavlou and Belsey, 2005; An, 2010; Beck et al., 2010; Leavy, 2010). Currently, nearly 50 mAb-related products, including several blockbuster drugs, are licensed to treat a variety of diseases in the US and Europe (Ecker et al., 2015).

Even though the development of mAb products has good prospects, the structure of mAbs are far more complex than those of small molecule drugs, including the primary structure, higher order structure, glycosylation and charge variants, etc. Besides having intrinsic heterogeneities, the mAbs introduced as part of bioprocess procedures are susceptible to further chemical modification and degradation (Pike, 1967; Haberger et al., 2014; Rosati et al., 2014). In addition, the binding activity, biological functions, and effector functions of mAbs are critical for their efficacy (Kaneko and Niwa, 2011; Overdijk et al., 2015; Kallewaard et al., 2016). Thus, to guarantee the quality and consistency of mAb productions, each step from the protein expression to the storage phase should be well controlled and characterized. Based on knowledge of the critical quality attributes of mAb, an analysis platform could be established to support the development of therapeutic mAbs and post-licensure life cycle management (Alt et al., 2016).

Moreover, as the patents of some mAb products expire, development of similar biotherapeutic products (SBPs) is becoming more and more popular (Yoo, 2014; Brinckerhoff and Schorr, 2015; Moorkens et al., 2016). Therefore, WHO’s *Guidelines on Evaluation of Similar Biotherapeutic Products* were adopted in 2009 by its Expert Committee on Biological Standardization. A stepwise approach was recommended to demonstrate the similarity between an SBP and the reference biotherapeutic product (RBP). These key principles serve well as a basis for establishing specific regulations for SBPs. However, due to the general complexity and heterogeneity of mAbs, comparability studies between SBP and RBP are challenging. Thus, an informal consultation of WHO was organized in 2015 to discuss these issues. All participants are agreed that the guidelines are still valid, valuable and applicable, but that further additional guidance was needed to evaluate biosimilar mAbs.

**Figure 1:**
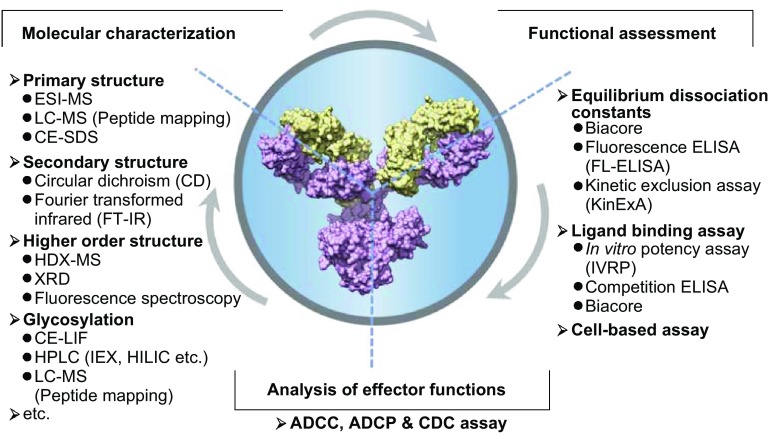
**Overview of the methods of molecular structural analysis and functional assessment**. As a complex macro-molecule protein, the molecular characteristics and functions are critical quality attributes for monoclonal antibodies. A series of physicochemical and biological methods are developed to evaluate these critical quality attributes carefully. In addition to the general molecular characterization, the function-associated analyses are listed in the right panel and bottom panel. The example monoclonal antibody is a representative IgG1 (PDB: 1IGY)

In summary, based on systematic pharmacological studies, potential mAb candidates could advance to the stage of development of investigational new drugs. The establishment of an efficient platform for quality analysis is critical for the development of mAb products, including biosimilar development. Thus, this review is focused on the analysis of molecular characteristics, potency (equilibrium dissociation constants, binding activity, and biological potency) and effector functions of mAb candidates (Fig. 1), and will also discuss the essential evaluations of mAbs as biosimilar therapeutics.

## Molecular Structure Analysis of Monoclonal Antibodies

With respect to molecular structure characterization of mAbs, structure, size, aggregation, heterogeneity, and glycosylation are critical attributes (Rosati et al., 2014). As a macromolecules, most therapeutic proteins are produced in live cells, followed by purification and formulation. Heterogeneities of mAb-based therapeutic proteins always appear in purified products. Post-translational modifications, degradation and other chemical modifications might occur during the preparation process and even during storage (Roque et al., 2004; Cohen et al., 2007; Shukla et al., 2007). With regard to the production of mAbs in living cells, protein folding and disulfide bond pairing are not always correct, and subsequent exposure to culture media and other stresses (such as pH, temperature, etc.) may cause further heterogeneity (Beck et al., 2013b).

Though purification processes will eliminate most unwanted molecules, a certain population of these molecules will remain with the main components. The following formulation step is thought to be beneficial to stabilization of therapeutic biologics and the storage of products. However, this process will sometimes augment the heterogeneity during the manipulations. Therefore, a series of physicochemical assays was established to analyze mAb characteristics, forming a favorable analysis platform that can be used to define well-characterized biologics.

### Primary structure

Complete and correct primary structure, DNA and amino acid sequences are the basis to ensure production of a desired mAbs. A mass spectrometer (MS) alone (Rosati et al., 2014) or coupled with reverse-phase high-performance liquid chromatography (Jung et al., 2014a) can be used to analyze the primary structure of mAbs with precise molecular weight. These tools can also be used to understand the amino acid sequence by peptide mapping of enzyme-digested protein, which can be used to analyze the lot-to-lot consistency (Fekete et al., 2013). In addition to high-performance liquid chromatography, capillary electrophoresis (CE) has been validated as an efficient separation method by pharmaceutical companies and regulatory agencies (Fekete et al., 2013; Zhao and Chen, 2014). Major advantages of CE include the ability to obtain separations within minutes while maintaining exceptional separation efficiency. Recently, a sheathless interface-based transient isotachophoresis CE-ESI-MS was used to characterize the complete amino acid sequences of mAbs in a single run (Gahoual et al., 2014). With this method, the primary structures of four different therapeutic mAbs were characterized in a robust manner with one injection.

### Higher-order structure

Even though primary structure is thought to determine the higher-order structures of proteins, post-translational modifications or the mispairing of disulfide bonds can dramatically affect the functions of proteins such as mAbs (Hattori et al., 2013; Filtz et al., 2014). Fourier transform infrared spectroscopy (FT-IR) and circular dichroism (CD) are two commonly used tools for the determination of secondary structure (Jung et al., 2014b; Telikepalli et al., 2014; Tsuchida et al., 2016). These methods can be used to determine percentages of α-helix, β-sheet, and random coils. Far UV CD can be used to examine the peptide backbone and estimate the secondary structure content of a protein. On the other hand, near UV CD spectra is generally used to characterize disulfide pairing and aromatic residues.

The tertiary structure, also known as the three-dimensional structure, is related to the functions of molecules. As reported, the change of tertiary structure of mAbs can be investigated using fluorescence spectroscopy (Huang et al., 2017; Wang et al., 2017). The degree of exposure of tryptophan will influence the maximal fluorescence emission wavelength. This indicator can be used to determine perturbations of mAbs’ tertiary structure (Vivian and Callis, 2001; Liu et al., 2014). Recently, some mass spectrometry-based methods have been developed to monitor the higher-order structures, including the native mass spectrometry, ion-mobility mass spectrometry, and hydrogen-deuterium exchange mass spectrometry (Huang and Chen, 2014; Thompson et al., 2014; Wei et al., 2014; Zhang et al., 2014; Terral et al., 2016). These methods are all rapid and sensitive.

### Aggregation

The dimerization and aggregation of therapeutic proteins are major challenges for the manufacture (Rosenberg, 2006; Roberts, 2014b; Singla et al., 2016; van der Kant et al., 2017), including the mAb-based biologics. Aggregation can dramatically influence the bioactivity of mAbs, and is generally irreversible (Roberts, 2014a). In addition, aggregates have the potential to cause side effects and increase the elimination rate due to their high immunogenicity (Ratanji et al., 2014). Therefore, the detection and characterization of aggregation is important. In general, several useful methods have been established to analyze aggregates, including high-performance size-exclusion chromatography, analytical ultracentrifugation, differential scanning calorimetry, dynamic light scattering, and asymmetric flow field-flow fractionation (Brych et al., 2010; den Engelsman et al., 2011; Zhang et al., 2012; Johnson, 2013; Krayukhina et al., 2013; Joshi et al., 2014; Pathak et al., 2014). Among these methods, high-performance size-exclusion chromatography is the most commonly used technique because of its ease of use. When coupled with other methods or instruments, such as multi-angle light scattering or mass spectrometry, the aggregation can be well characterized (Fekete et al., 2014; Marassi et al., 2014). In addition, with the use of hydrogen-deuterium exchange mass spectrometry, the intermolecular binding sites can be mapped, which is beneficial for further improving the design of molecules (Iacob et al., 2013; Moorthy et al., 2014).

### Heterogeneity

#### Charge variants

Besides the above mentioned sources of heterogeneity, certain analytical procedures could introduce stress challenging molecular stability, and thus cause assay-induced heterogeneity. To avoid this artificial heterogeneity, a solution-based method (Cao et al., 2016) that can detect proteins in a native state is preferred, especially for assessment of heterogeneity associated with charge variants.

CE-based isoelectric focusing or capillary isoelectric focusing (CIEF) is a potent method to analyze the charge variants of mAb-based products (Hunt et al., 1998; Hong et al., 2014; Salmanowicz et al., 2014; Suba et al., 2015). This method focuses the different mAb variants based at the pH points where the total net charge is zero, also known as isoelectric points. The analytes are mainly focused in the CIEF gel-based solution without resin interaction and surface adsorption, conditions that are less intrusive to the molecules (Shimura, 2002). This method can be used to analyze both native and denatured proteins. However, CIEF requires specific gels, ampholytes and pI markers, all of which are costly reagents. Further, this method is time-consuming, commonly taking 30 to 40 min for focusing and separating. Thus, a more convenient method, capillary zone electrophoresis (Moritz et al., 2015), has been established to supplement CIEF, especially in the early stage of clonal selection and process optimization (Jorgenson and Lukacs, 1983; He et al., 2011). Capillary zone electrophoresis can assess the samples rapidly and with simple sample preparation as compared with other charge variant analysis methods. More recently, several capillary-derived methods were established to improve throughput of charge variants analysis. For example, NanoPro technology coupled with photochemical immobilization and chemiluminescence technology could be used for high throughput measurement of the charge heterogeneity of mAb products (Michels et al., 2012).

Ion exchange chromatography (IEX), including anion IEX (AIEX) and cation IEX (CIEX), is the other commonly used method to analyze the charge heterogeneity (Talebi et al., 2014). Unlike IEF, IEX can distinguish differences in the surface charge of molecules. Therefore, the results gained from IEX can reflect structural information (Kluters et al., 2016). In comparison to the IEF, IEX better tolerates the buffer matrix through the process of adsorption and desorption to resin. With the wide use of mAb products, the production must be more efficient. Membrane-based IEX can help to meet this demand (Knudsen et al., 2001).

#### Size associated heterogeneity

Size distribution is important for the safety and efficacy of mAb products. Size changes are always associated with enzymatic (or nonenzymatic) cleavage or mispaired and incomplete formation of disulfide bonds. Sodium dodecyl sulfate polyacryl-amide gel electrophoresis (SDS-PAGE) and high-performance size-exclusion chromatography (Tous et al., 2005) are the major methods used to assess the size heterogeneity of mAbs. With high/ultra-high performance size-exclusion chromatography, native or denatured samples can be assessed with results indicating whether association is covalent or noncovalent (Yang et al., 2015). SDS-PAGE, with or without the use of reducing agents can shed light on the situation of covalent linkages. In addition to high/ultra-high performance size-exclusion chromatography and SDS-PAGE, other methods have been established to analyze the size distribution of mAbs with increasing frequency. These methods include dynamic light scattering (Zhou et al., 2015), analytical ultracentrifugation, and field-flow fractionation.

#### Glycosylation assessment

Addition of different oligosaccharides, glycosylation will influence the effector functions of mAbs dramatically. As reported, N-linked glycosylation is the most common type found in mAb products. The absence of these oligosaccharides has no effect on the binding ability but has a profound effect on mAb effector functions (Wright and Morrison, 1997; Arnold et al., 2007). Thirty-two unique oligosaccharides may be added to Asn297, and subsequent random pairing of heavy chains could generate almost 500 glycoforms (Jefferis, 2009). PNGase F is a commonly used reagent to release the oligosaccharides from the heavy chain of mAbs. Subsequent MALDI-TOF was used to assess the released N-glycosylation. This method employs dihydroxybenzoic acid as a matrix to measure the mass of free glycans.

CE is another convenient method to monitor Asn297 glycosylation. Without release of oligosaccharides, CE-SDS can be used to rapidly analyze the glycosylation states of mAbs under both non-reducing and reducing conditions (Rustandi et al., 2008b; Kotia and Raghani, 2010; Esterman et al., 2016). Under reducing conditions, the electropherogram usually contains two peaks, representing light chains and heavy chains. However, in some samples, the heavy chain peak may contain a minor one, which represents a non-glycosylated heavy chain (Rustandi et al., 2008a). CE-SDS can analyze samples rapidly and with high sensitivity. In addition to detection by diode array detector, CE can be used to analyze released oligosaccharides through detection using laser-induced fluorescence. Released glycans can be coupled with a fluorophore, called APTS, through a sodium cyanoborohydride mediated cross-linking reaction. The stoichiometry of the labeling reaction is one APTS molecule per molecule of oligosaccharide. With the use of quantitation control (G22) or a labeled glucose ladder standard (G20), this method can be used to determine the size of glycans and provide quantitation and mobility characterization of the released oligosaccharides.

## Functional assessment

In addition to the abovementioned attributes, function is another critical attribute for mAb-based biologics. Before clinical trials, candidate molecules must be tested in animal models. Histological methods can be used to demonstrate the function of molecules. In addition to histological studies, ligand-binding ability, cell-based function, and affinity constant of mAb-based products are also important. The following section will focus on methods or technologies used to characterize these attributes.

### Equilibrium dissociation constants

Antibody-antigen (Ab-Ag) interaction is critical for the function of mAbs. As a key parameter of Ab-Ag interaction, the equilibrium dissociation constant, also known as the *K*_*D*_ value, can be used to predict the interaction status under certain conditions (Azimzadeh and Van Regenmortel, 1990; Sirin et al., 2016). When the total concentrations of Ab and Ag are higher than the *K*_*D*_ value, most binding partners exist in the associated form. Otherwise, only a small proportion of Ab-Ag bind together to form a complex. Based on different mechanisms, SPR-based technology (Schuck, 1997; Gopinath and Kumar, 2014), fluorescence ELISA (FL-ELISA) (High et al., 2005), and kinetic exclusion assays (KinExA) (Bee et al., 2012) are commonly used to determine the *K*_*D*_ value.

BIAcore is widely used to monitor the real-time interaction of biomolecules. This method has been used during early or advanced stages of the development process of antibodies. A special optical biosensor is applied to measure the change of refractive index when ligands bind. This response is proportional to the mass that binds at the surface of the biosensor. The process is performed in solution, so no interference is introduced during the assay.

Although the solution affinity can be determined with SPR-based technology, *K*_*D*_ values lower than 100 pmol/L are difficult to measure. As reported, fluorescence-based ELISA and KinExA technology can be used to address this issue. FL-ELISA is a convenient and sensitive method for the quantitation of low level analytes. Compared to colorimetric-based assays, the sensitivity of FL-ELISA is found to be enhanced 5 to 10 fold. In addition, its detection limit of *K*_*D*_ values has been demonstrated to be as low as 10 pmol/L (High et al., 2005). This solution-based method can also measure the dissociation constant without modifications or surface adsorption. KinExA is a technique for measuring the concentration of one of the reactants in a two-phase reversible reaction mixture without perturbing the equilibrium of the solution-bound components. The assumption underlying kinetic exclusion is that the time of contact between the mixture and the solid phase is sufficiently short that there is insufficient time for significant dissociation of the solution-bound component to occur. Thus, the captured portion of the free component provides a direct measure of the amount free at equilibrium.

### Ligand-binding assay (*in vitro* potency assay)

Unlike the *in vivo* assay, the *in vitro* binding activity is used as a surrogate method to analyze the mAb candidates. A stable and soluble antigen is needed to represent the *in vivo* target of mAbs. In addition, critical epitopes that are recognized by the mAb should be well defined with the use of hydrogen-deuterium exchange mass spectrometry or other methods. As a surrogate method, the ligand-binding assays are useful in the early development phase and even in the life cycle management phase.

**Figure 2:**
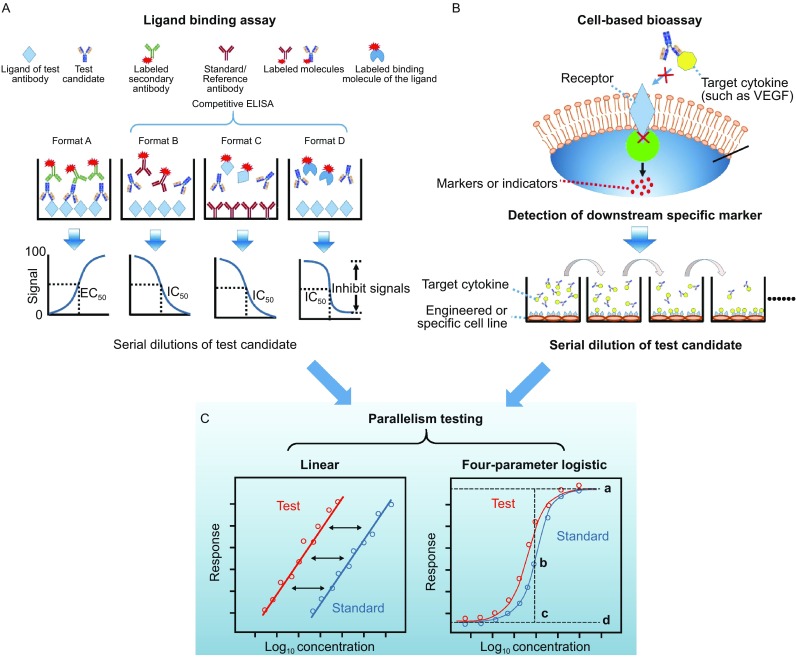
**Schematic diagrams of ligand-binding assays and cell-based potency assay**. (A) Four different types of ELISA-based ligand-binding assays (Biacore assays can be designed in a similar way). Format A is a direct way to evaluate the binding activity to the intended receptor. Formats B and C are both competitive ELISA; Format C is highly preferred for purified IgG since there are no subsequent wash cycles for the test antibody, unlike in the other three types. Format D is a ligand-blocking assay in the form of a competitive ELISA. (B) Cell based bioassay. The therapeutic mAbs are generally target cytokines or cell-surface receptors (In this diagram, the mAbs target cytokines as an example). Based on the understanding of the mechanism of action of mAbs, an engineered or specific cell line should be developed, and the marker should be defined. For example, human umbilical vein endothelial cells or the NFAT-RE-luc2P/KDR HEK293 cell line are used to assess the mAbs of VEGF, and the calcineurin-NFAT pathway could be used as the key marker of VEGF-mediated angiogenesis. (C) Parallelism test between data sets for test articles and the reference. Data obtained from the ligand binding assays and cell-based assay was recommended to analysis by using parallelism tests, including linear model and four-parameter logistic model, to estimating sample potency relative to a given standard. The symbols “a” in four-parameter logistic model represent upper asymptote, “b” represent slope parameter, “c” represent EC_50_, “d” represent lower asymptote

ELISA and surface plasmon resonance (SPR)-based technology are two most popular methods for ligand-binding analysis. Based on understanding of the epitope of the target antigen, four different forms of ELISA assay can be used to evaluate the binding ability of mAbs. In general, SPR-based assays can be designed in a similar way. With regards to the four forms of ELISA, evaluation of the relative ED_50_ (the effective concentration needed for 50% of maximal binding) is preferred during the early development of mAb products since only a few antibody molecules are available at this stage. With an appropriate antigen coated on 96-well plates (called Format A, Fig. 2A), the test antibody can bind to the surface specifically, following by a wash. The test antibody that binds the antigen can then be captured by a labeled secondary antibody, and subsequently assessed through the detection of the optical density (OD). With serial dilution, the binding curve reflecting the binding ability of mAb products is obtained, and the EC_50_ value can be derived by four-parameter logistic fit (Fig. 2C) with the use of GraphPad Prism 5.0 (GraphPad Software, San Diego, CA). With the use of standards, the ratio of EC_50_ values (or relative EC_50_, rEC50) can be obtained by the EC_50_ value of the standard over the EC_50_ value of test mAb. This value would indicate the binding activity of the test mAb compared to that of the standard. The greater the rEC_50_ value, the higher the binding activity.

In the later stages of drug development, solution competitive ELISA is highly preferred for purified candidate molecules, more subtle differences can be detected in this method. Three types of competitive ELISA have been developed to detect the binding activity of mAb molecules. In the first format, labeled Format B in Fig. 2A, specific antigens are coated on the surface of 96-well plate. Serially diluted test mAbs are premixed with a constant labeled standard. There is a competition of binding ability between the test mAb and standard. The second competitive ELISA, labeled Format C in Fig. 2A, uses a standard mAb as coating. A serially diluted test mAb is premixed with a labeled antigen, with an equal amount in the different wells. Both assays are useful to analyze the quality of mAbs through the measurement of IC_50_ values. In addition, if an antigen-specific ligand could be achieved, a ligand blocking assay-based competitive ELISA could be established. The ligand blocking assay, labeled Format D in Fig. 2A, is similar to Format B, but the labeled antibody is replaced with a labeled binding molecule of the ligand. The mode of action, affinity, and footprint can be elucidated with the use of the ligand-blocking assay. However, a soluble and labeled ligand is essential for this assay format.

In summary, if the mechanism of action of therapeutic mAbs expected to be binding activity to a specific ligand, the binding assay can be used as lot release assay along with the cell-based assay during clinical development phases. Upon the product licensure, the database on potency assays and the inputs from regulatory agents should be taken into consideration as to whether one assay can be chosen over the other as a long term lot release assay post licensure. Both indirect ELISA (Format A) and competitive ELISA (Format B to D) could be developed easily and efficiently. Compared these two types of ELISA, competitive ELISA is preferred at a later stage of therapeutic antibody development since it could analyze solution activity of the test molecules (or the drug molecule) in a quantitative manner. Especially in Format C, the analytes in solution with native conformation will not be subjected to subsequent wash cycles in which certain interactions could be disrupted.

### Cell-based potency analysis

Even though the ligand-binding assay can be performed readily with desirable precision and accuracy, the mechanism of action could involve downstream events post ligand-binding. Thus, measuring the binding activity alone may not reflect the mAb potency in a faithful manner, and this practice may have some regulatory risk. For example, mAbs with different targets generally induce an early response (signaling pathway) or a late response (proliferation, cytokines). Therefore, the product potency should be evaluated by cell levels based on the understanding of the mechanism of action of the mAb (Fig. 2B). A downstream marker (early response, late response or cell adhesion, etc.) that is normally inhibited with the use of the mAb should be defined. Through quantitative or qualitative analysis, the biological activity could be demonstrated by comparison with controls. For example, to evaluate the function of a vascular endothelial growth factor (VEGF)-specific antibody (such as bevacizumab), human umbilical vein endothelial cell was generally used to establish the biological potency assay (Papadopoulos et al., 2012). However, based on the knowledge that VEGF targets VEGF receptor-2 (VEGFR-2, expressed mainly on vascular endothelial cells) and subsequently activates calcineurin-nuclear factor of activated T cell (NFAT) signaling, a reporter gene assay (using engineered NFAT-RE-luc2P/KDR HEK293 cell line) was developed to assess the potency of VEGF-specific mAb. Except for the cytokine-specific mAbs, many therapeutic antibodies recognize cell surface receptors, such as the rituximab which targets CD20. One of its mechanism of actions is the induction of apoptosis of CD20^+^ cells. To evaluate the apoptosis, Annexin V-FITC could be used to indicate the mAb-induced externalization of phosphatidylserine on specific cells, and dead cells could be stained with propidium iodide.

In summary, the establishment of cell-based potency assays is useful to indicate the biological activity of test antibodies. Functional cell-based assay could better reflect the mechanism of action of a therapeutic mAb than a ligand binding assay (Hansel et al., 2010; Tada et al., 2014). Moreover, compared to binding assay, cell-based bioassay could detect the impact of chemical modifications, such as deamidation in the complementarity-determining region or the Fc region of the molecule on its potency. Therefore, cell-based assays should be primarily chosen for product characterization, even for lot release, during the clinical development of mAb-based drug and post licensure life-cycle management.

### Assessment of effector function

Many mAb-based products target soluble receptors or a cytokines on the cell surface, thereby triggering complex downstream signaling events. The Fab fragment is mainly associated with binding specificity, while the Fc portion is critical for the function of IgG at the cell level and for its metabolic fate. For candidate molecules, complement activation and other effector functions are important, and should be well-studied during development. These effector functions include antibody-dependent cellular cytotoxicity (ADCC), complement-dependent cytotoxicity (CDC), and antibody-dependent cell-mediated phagocytosis (ADCP).

**Figure 3:**
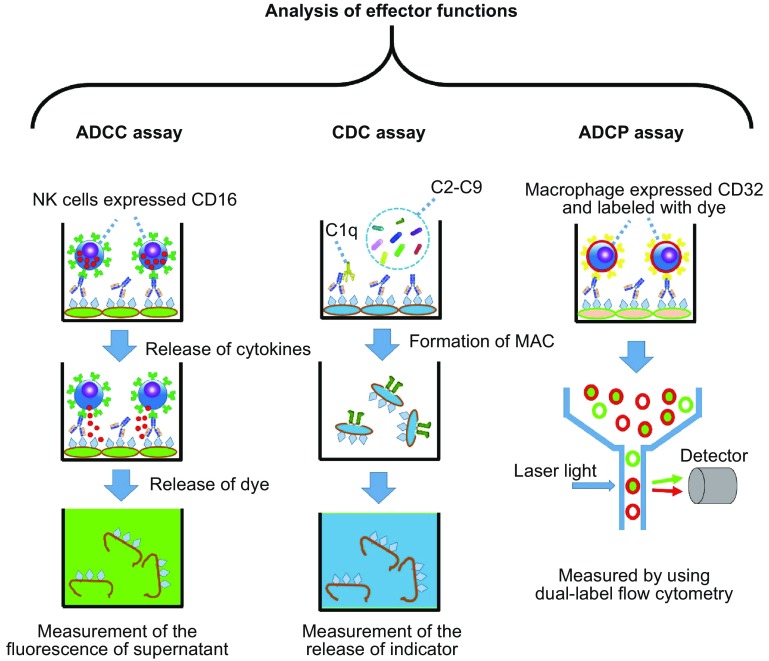
**Analysis of effector functions**. For the ADCC assay (left panel), target cells are labeled with an indicator (such as calcein) and opsonized by using the test antibody at the indicated final concentration, effector cells (purified NK cells or peripheral blood mononuclear cells) are added in an appropriate ratio to target cells, and the final fluorescence intensity of the supernatant is measured. The procedure of the CDC assay (middle panel) is similar to the ADCC assay, except for the use of a complement to replace the effector cells. For the ADCP assay, target cells and the macrophage (differentiated by purified monocytes) were first labeled with fluorescent dyes and opsonized with the test antibody at indicated final concentration, after which the fluorescence was measured with a dual-label flow cytometry

The assessment of effector functions is important for the development of original mAb candidates and biosimilar molecules (Beck et al., 2013a). However, the establishment of an ADCC/CDC assay should be performed according to the characteristics of the mAbs (Cheng et al., 2014). For example, for Amgen’s candidate molecule ABP501, which is a biosimilar of adalimumab, an assay used Chinese hamster ovary (CHO) target cells (CHO M7, Amgen) to evaluate the induction of ADCC and CDC. CHO M7 expresses a cell-surface-displayed non-cleavable TNF-α. As described by Liu and colleagues, calcein-acetoxymethyl (AM) was used as an indicator to evaluate the level of ADCC (Fig. 3, left panel) during co-incubated with effector cells (human CD16 stably transduced NK92-M1) (Liu et al., 2016). The CDC assay is similar to the ADCC assay, but the NK cells are replaced with baby rabbit complement, as shown in Fig. 3 (middle panel). For the ADCP assay, macrophage (derived from the purified monocytes) and target cells are labeled with different dyes, and when the test antibodies exist, the ADCP can be detected with the use of dual-label flow cytometry (Fig. 3, right panel).

For the potency assessment, many mAbs do not require a functional bioassay for phase I, but many antibody-drug conjugates may require a bioassay. This indicates that even though the ligand binding assays and cell-based assays are relevant, the former may provide the best precision for process and formulation development, while the latter are good for indicating if changes are either enhanced or tolerated in a biological system.

## Evaluation of Monoclonal Antibodies as Similar Biotherapeutic Products

According to WHO guidelines (*Guidelines on Evaluation of Similar Biotherapeutic Products*), the quality similarity of an SBP and RBP should be confirmed before moving forward to comparative non clinical and clinical studies. To improve the credibility of the results, multiple batches of SBP and RBP should be used. The minimum number of batches that should be tested depends on the variability of the reference product and on the assay variability. Additionally, to obtain unequivocal results, the methods used should be scientifically valid and appropriate.

Unlike many other proteins, mAbs are glycoproteins with complex structures and intrinsic heterogeneities. Therefore, the comparability studies should be well designed, including both non-clinical and clinical studies. In terms of non-clinical development, a stepwise approach is recommended. The *in vitro* studies, which are sufficiently sensitive and specific to observe the differences in quality attributes, should be conducted first. In addition, based on these results, a decision about which *in vivo* study is required can be made before initiating clinical trials. The following discussion focuses on the several considerations for non-clinical studies and clinical studies recommended by WHO.

First, *in vitro* studies are paramount for non-clinical biosimilar comparability evaluation. A whole spectrum of pharmacological and toxicological aspects should be considered during the selection of *in vitro* assays. Relevant assays should include binding studies, functional studies, and studies of biological activities. However, as recommended by the ICH S6 (R1) guideline, tissue cross-reactivity studies with mAbs should not be used to assess the comparability because these studies are insufficiently powerful to detect the subtle differences in critical quality attributes. If the results obtained from the quality comparability studies and non-clinical *in vitro* studies are not satisfactory, an *in vivo* animal study should be considered to provide complementary information. The *in vivo* assays should be designed based on the needs of the residual uncertainty about the quality, and maximize the information obtained. When a suitable model is available, pharmacokinetics, pharmacodynamics, safety, immunogenicity, local tolerance and other studies can be performed to further evaluate the SBP and RBP.

When a clinical comparability evaluation is needed, the main purpose is to confirm that any residual quality-related uncertainty will not introduce clinically meaningful differences. Clinical pharmacokinetics and pharmacodynamics studies are generally needed to monitor the impact of the formation of anti-drug antibodies. The last step is to confirm the comparability of the efficacy of the SBP. In general, a randomized, double-blinded, and powered clinical efficacy study should be performed.

Based on these principles and approaches, the regulatory agency could further set up their own laws and regulations, which are important to ensure that the biosimilar therapeutic mAb products can be well characterized without unneeded effort.

## Conclusion and Discussion

Due to the complexity and intrinsic heterogeneity of monoclonal antibodies and antibody-related products, extensive biophysical, biochemical, biological, immunochemical, and immunological characterizations should be carefully conducted. This review focused on the methods and technologies that are used to characterize mAb-based candidates during preclinical and clinical studies. With this quality-associated analytical platforms, the primary, secondary, tertiary, and quaternary structures, heterogeneity, affinity, ligand-binding ability, glycan structures, and other characteristics of mAbs would be quantitatively evaluated. These values from the quantitative methods provide a comprehensive and matrixed package for multiple lots of products accumulated during the preclinical and particularly clinical developmental phases. Such a working database on the product would enable future comparability exercise in support of process upgrade or scale up to support expanding markets or alternative production facility.

In addition to traditional mAb products, there are other forms of mAb-based biologics such as antibody-drug conjugations and bi-specific antibodies. The principles of analysis for these molecules are generally consistent with those of the traditional mAbs, but there are several specific attributes that must be designed, such as the stoichiometry between IgG and the drug or the “drug-to-antibody ratio” and “drug distribution” for the antibody-drug conjugations (Hamblett et al., 2004; Wakankar et al., 2011), among others.

As one can imagine, with the development of analytical technology, the characterization of mAbs will becomes more precise, systematic and even more important, not only for the development of future mAb products but also for evaluation of biosimilar mAbs. Nevertheless, the concept of quality by design (QbD) should be used during the development of therapeutic mAbs (Finkler and Krummen, 2016; Kelley, 2016). Based on the pre-establishment of target product quality profile, developers could identify the critical quality attributes by the design of experiments, and then determine the workspace in bioprocessing with the critical process parameters. The combination of different analytical methods is used to monitor the product quality in different stages of the bioprocessing as well as in the formulated products. Database from manufacturing and from stability testing should be carefully maintained to ensure the consistency in the manufacture process (such as in an event of a scale up or tech transfer to a different manufacturing facility) and the product stability profiles of the licensed therapeutic mAbs.
